# The Role of PPAR-δ in Metabolism, Inflammation, and Cancer: Many Characters of a Critical Transcription Factor

**DOI:** 10.3390/ijms19113339

**Published:** 2018-10-26

**Authors:** Yi Liu, Jennifer K. Colby, Xiangsheng Zuo, Jonathan Jaoude, Daoyan Wei, Imad Shureiqi

**Affiliations:** 1 Department of Gastrointestinal Medical Oncology, The University of Texas MD Anderson Cancer Center, 1515 Holcombe Boulevard, Unit 426, Houston, TX 77030-4009, USA; yliu25@mdanderson.org (Y.L.); jkcolby@mdanderson.org (J.K.C.); xzuo@mdanderson.org (X.Z.); Jjaoude@mdanderson.org (J.J.); 2 Department of Gastroenterology, Hepatology, and Nutrition, The University of Texas MD Anderson Cancer Center, Houston, TX 77030, USA; dwei@mdanderson.org

**Keywords:** PPAR-δ, β-oxidation metabolism, inflammation, cancer

## Abstract

Peroxisome proliferator-activated receptor-delta (PPAR-δ), one of three members of the PPAR group in the nuclear receptor superfamily, is a ligand-activated transcription factor. PPAR-δ regulates important cellular metabolic functions that contribute to maintaining energy balance. PPAR-δ is especially important in regulating fatty acid uptake, transport, and β-oxidation as well as insulin secretion and sensitivity. These salutary PPAR-δ functions in normal cells are thought to protect against metabolic-syndrome-related diseases, such as obesity, dyslipidemia, insulin resistance/type 2 diabetes, hepatosteatosis, and atherosclerosis. Given the high clinical burden these diseases pose, highly selective synthetic activating ligands of PPAR-δ were developed as potential preventive/therapeutic agents. Some of these compounds showed some efficacy in clinical trials focused on metabolic-syndrome-related conditions. However, the clinical development of PPAR-δ agonists was halted because various lines of evidence demonstrated that cancer cells upregulated PPAR-δ expression/activity as a defense mechanism against nutritional deprivation and energy stresses, improving their survival and promoting cancer progression. This review discusses the complex relationship between PPAR-δ in health and disease and highlights our current knowledge regarding the different roles that PPAR-δ plays in metabolism, inflammation, and cancer.

## 1. Introduction

Peroxisome proliferator-activated receptor-delta (PPAR-δ, also known as PPAR-β) is a member of the PPAR subgroup in the nuclear receptor superfamily. PPARs act as ligand-activated transcription factors that regulate important cellular metabolic functions [1]. Although PPAR-δ is ubiquitously expressed, its expression level in different tissues varies depending on cell type and disease status [2,3,4,5]. Homozygous knockout of murine *Ppard* through constructs targeting exon 4, which codes for the DNA binding domain, leads to embryonic lethality or impaired growth, which indicates that PPAR-δ plays a fundamental role in embryo development [6,7].

Details of PPAR structure and signaling mechanisms have been reviewed in detail in Reference [8] and will only be discussed briefly here. The characteristics of PPAR ligand-binding domains (LBD) allow for interaction of a broad range of potential ligands, including many lipid and lipid-like molecules [8]. Natural ligands for PPAR-δ include polyunsaturated fatty acids (PUFA, e.g., arachidonic and linoleic acid)) and their metabolites (e.g., prostacyclin/PGI_2_, 13*S*-hydroxyoctadecadienoic acid (13*S*-HODE), and 15*S*-hydroxyeicosatetraenoic acid (15*S*-HETE)) [9,10,11,12]. Although PPAR-δ has a narrower LBD relative to PPARs-α and-γ, binding pocket characteristics allow potential interaction with a variety of ligands, albeit many appear to bind at relatively low affinities [13]. While many potential endogenous ligands have been suggested in the literature, there is still some uncertainty about the physiological significance [14]. Selective ligands targeting PPAR-δ have also been developed, although none have been approved for clinical use to date [14,15].

PPAR signaling can be regulated in multiple ways, with outcomes depending upon whether PPAR and its binding partners are bound by ligand or not, ligand type (agonist, antagonist, partial agonist, etc.), and concentration as well as the availability of various coactivators or repressors [8]. The delivery of natural PPAR-δ ligands is facilitated by fatty acid transport proteins (FATPs) and fatty acid translocase (FAT, also known as CD36), which aid in import of extracellular lipids into the cell [16,17] and fatty-acid-binding proteins (FABPs), which transport cytoplasmic lipids within the cell [18,19]. Although most FABPs can bind a number of different lipids, it is unknown whether there is any selectivity in terms of the ligands FABP shuttles to PPARs [18,20,21]. In relation to PPAR-δ, FABP5 (also known as K-FABP or E-FABP) appears to be important for transport of lipid ligands to the nucleus [22]. Interestingly, FABP5 expression largely parallels that of PPAR-δ, and interaction between the two appears to be important in both normal and disease states, including many cancers [19]. Although a more detailed discussion is beyond the scope of this review, the interrelationship between the PPARs, their endogenous ligands, and various lipid transport proteins is complex, and several of these transport proteins are known transcriptional targets of PPARs (reviewed in References [16,19]).

Activation of PPARs by their ligands has been discussed in detail elsewhere and will be described only briefly here [8,23,24]. PPAR-δ activation requires interaction with various partners in the nucleus to transcriptionally regulate gene expression. Like other PPARs, PPAR-δ heterodimerizes with the retinoid X receptor (RXR) to activate or repress expression of downstream target genes by binding to PPAR response elements (PPREs) in their promoters [25,26]. In the absence of ligand binding, PPAR-RXR complexes are associated with corepressive factors and histone deacetylases that prevent transcriptional activation. Binding of an activating ligand to PPAR-δ leads to conformational changes that release corepressors and allow binding of coactivators [8,27]. In addition, PPARs can also engage in transrepression of other transcription factors. For example, in its unliganded state, PPAR-δ has been shown to form a complex with the transcription factor BCL-6, which prevents BCL-6 from repressing proinflammatory cytokine genes; therefore, this interaction promotes inflammation [28,29]. Conversely, binding of PPAR-δ agonist leads to disruption of the complex, and BCL-6 is freed to repress gene expression [28]. PPAR-δ has also been reported to interact with other transcription factors, such as β-catenin or NF-κB, to regulate gene expression [30,31].

Accumulating evidence has demonstrated that PPAR-δ can have distinct roles depending on the context (e.g., healthy vs. diseased, specific type of disease). While PPAR-δ allows normal cells (e.g., muscle cells and pancreatic cells) to better cope with adverse nutrient and energy pressures, PPAR-δ overexpression or hyperactivation can lead to promotion of inflammation and tumorigenesis. We will address some of the known discrepancies concerning PPAR-δ’s putative roles in metabolism, inflammation, and cancer in this review.

## 2. Metabolic Regulation by PPAR-δ

Modulation of cellular energy consumption is a major function of PPAR-δ. In muscle cells, ligand activation of PPAR-δ switches energy production from glycolysis to fatty acid oxidation as an alternative energy source, which can enhance muscle endurance [32]. In skeletal muscle cells, PPAR-δ activation by fatty acids increases fatty acid uptake and catabolism via β-oxidation [33]. Genetically targeting PPAR-δ overexpression in skeletal muscle cells increases succinate-dehydrogenase-positive muscle fibers with enhanced fatty acid oxidative capabilities and leads to an overall decrease in body fat [34]. Treatment of insulin-resistant obese monkeys with the PPAR-δ ligand GW501516 increased serum high-density lipoprotein cholesterol while decreasing low density lipoprotein, fasting triglycerides, and insulin [35]. Similarly, in vivo and in vitro transcriptome analyses of rodent muscle showed that PPAR-δ regulated downstream target genes required for fatty acid transport, β-oxidation of fatty acid, and mitochondrial respiration. Transgenic activation of PPAR-δ in mouse adipose tissues upregulated the expression of genes involved in fatty acid β-oxidation and energy dissipation via uncoupling of fatty acid oxidation and ATP production; these effects were observed in both *ob/ob* (mice homozygous for the spontaneous obese mutation (*ob*) in the leptin (*Lep*) gene) and wild-type (WT) mice on high-fat diet [36]. In contrast, PPAR-δ knockout mice were more prone to high-fat-diet-induced obesity [36]. A recent report showed induction of fatty acid oxidation in intestinal stem cells after a 24h fast; this effect was observed in both young and old mice and was mediated by the PPAR-δ target gene carnitine palmitoyltransferase 1 (*Cpt1a*) [37].

In addition to its effects on fatty acid oxidation, PPAR-δ leads to improved blood glucose homeostasis through a number of mechanisms. PPAR-δ is strongly expressed in pancreatic islet beta cells, promoting insulin secretion [38,39]. It is also protective against insulin resistance through effects on hepatic and peripheral energy substrate utilization [40,41,42]. Treatment of *ob/ob* mice, which have a genetic predisposition to obesity and diabetes, with GW50516 attenuated the ability of high-fat diet to induce obesity and insulin resistance and improved diabetes [43].

**Figure 1:**
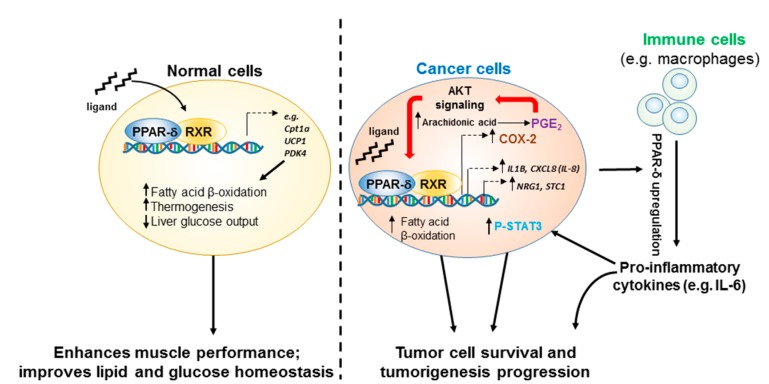
Ligand-dependent actions of PPAR-δ in normal versus cancer cells. Binding of PPAR-δ agonists in normal cells (**left**) leads to the upregulation of genes associated with a switch to using fatty acids as an energy source (increased β-oxidation). It is also associated with systemic improvements in serum glucose regulation through effects on multiple tissues, including pancreas, adipose, liver, and muscle. In cancer cells (**right**), this capacity for PPAR-δ to promote use of fatty acid substrates as an energy source can enhance cell survival and proliferation under harsh metabolic conditions frequently found in tumors. In addition, both COX-2 and PI3K/AKT signaling pathways are often upregulated in tumor cells. Interaction of activated PPAR-δ with these key signaling hubs leads to establishment of a feed-forward circuit promoting cancer development and progression through upregulation of additional factors that enhance neoplastic processes in cancer cells themselves as well as noncancer cells (e.g., tumor-associated macrophages) that make up the tumor microenvironment. See text for additional details.

These salutary PPAR-δ functions in normal cells are thought to protect against metabolic-syndrome-related diseases, such as obesity, dyslipidemia, insulin resistance, hepatosteatosis, and atherosclerosis [44,45]. Therefore, highly selective synthetic PPAR-δ agonists (e.g., GW0742 [46], GW501516 [35]) were developed and tested clinically. However, improving cellular tolerance to an inhospitable metabolic microenvironment could also promote the survival of cancer cells (Figure 1). For example, overexpression of PPAR-δ was shown to improve breast cancer cell survival during low-glucose or hypoxic cell culture conditions through multiple mechanisms (e.g., enhanced antioxidant signaling, AKT/protein kinase B activation), and increased cell survival was inhibited with PPAR-δ antagonists [47]. Other studies have demonstrated that PPAR-δ promotion of fatty acid oxidation can lead to increased ATP production, contributing not only to the survival of breast cancer cells [48] but also other cancer cells, such as chronic lymphocytic leukemia cells [49]. Concerns regarding the potential protumorigenic effects of PPAR-δ have led to halting of the clinical development of PPAR-δ agonists [50,51]. 

## 3. PPAR-δ in Inflammation-Related Diseases

Many studies have revealed that PPARs are involved in regulation of inflammation. Initially, PPARs were generally believed to have anti-inflammatory functions, and current research has more clearly defined such roles for PPAR-α and PPAR-γ [52,53]. PPAR-δ’s relationship with inflammation seems to be much different and still needs to be fully elucidated. In some contexts, PPAR-δ has been reported to have anti-inflammatory functions. For example, it was reported that the selective PPAR-δ agonist GW0742 alleviated inflammation in experimental autoimmune encephalomyelitis (EAE), while knockout of PPAR-δ aggravated EAE severity [54,55]. PPAR-δ’s antidiabetic functions also appear to be associated with reduced inflammatory signaling. In a rat model of type 2 diabetes, GW0742 was shown to reduce the proinflammatory cytokines tumor necrosis factor-α (TNF-α) and monocyte chemoattractant protein-1 (MCP-1) in liver tissues, in conjunction with reduced hepatic fat accumulation [56]. GW0742 was also shown to inhibit streptozotocin-induced diabetic nephropathy in mice through a reduction of inflammatory mediators, including MCP-1 and osteopontin [57]. In addition, a more recent study using both the *db/db* (homozygous for the spontaneous *db* mutation in the leptin receptor gene (*Lepr*)) and high-fat-diet-induced obese diabetic mouse models showed that PPAR-δ is a key mediator in exercise-induced reduction of vascular inflammation; PPAR-δ knockout mice did not exhibit this improvement with exercise [58].

In contrast to the reports mentioned above, PPAR-δ signaling appears to promote inflammation in other contexts. PPAR-δ expression is increased in patients with psoriasis, a common immune-mediated disease primarily affecting the skin [59]. In a transgenic mouse model, induction of PPAR-δ activation in the epidermis led to development of a psoriasis-like skin condition, which was correlated with increased IL-1 signaling and phosphorylation of STAT3 [59]. PPAR-δ signaling may also promote inflammation in some forms of arthritis. Mesenchymal stem cells (MSCs) have immunomodulatory properties that can limit inflammation [60]. In a collagen-induced mouse model of arthritis, mice receiving MSCs with reduced PPAR-δ activity (MSCs harvested from PPAR-δ knockout mice or WT PPAR-δ MSCs pretreated with the PPAR-δ antagonist GSK3787) had better suppression of inflammatory immune responses, leading to improvements in arthritis scores [61]. In the same study, inhibition of PPAR-δ with GSK3787 in human MSCs enhanced their ability to limit proliferation of peripheral blood mononuclear cells in coculture experiments [61].

## 4. Modulation of Inflammatory Actions in Immune Cells

Mechanistically, fatty acid oxidation is a central metabolic factor impacting immune cell differentiation and activation; for example, the behavior of inflammatory and immunosuppressive T cell and macrophage subsets can be affected by the balance of fatty acid oxidation and synthesis [62,63,64]. PPAR-δ’s roles as a fatty acid sensor and regulator of immune responses suggest a novel role for this receptor in metabolic modulation of immune cell differentiation and activity.

PPAR-δ appears to regulate inflammation through its effects on immune cells, particularly macrophages. Deletion of *Ppard* in liver-specific macrophages (Kupffer cells) led to reduced sensitivity to interleukin-4 and, therefore, impaired polarization to an alternative (M2-like) state with reduced inflammatory potential relative to M1-like macrophages [65]. This failure of differentiation to the M2 state ultimately led to hepatic dysfunction and insulin resistance, which was associated with altered fatty acid metabolism [65]. In another study using macrophage-specific PPAR-δ knockout mice, the deletion of *Ppard* was shown to decrease the phagocytosis of apoptotic cells and inhibit anti-inflammatory cytokine production by macrophages, leading to increased susceptibility to autoimmune kidney disease [66]. However, other evidence has revealed PPAR-δ’s modulation of inflammation may be even more complex. In primary human monocyte-derived macrophages (MDMs), PPAR-δ ligands were reported to repress inflammation-associated NF-κB and signal transducer and activator of transcription 1 (STAT1)-targeted genes (e.g., *CXCL8* (IL-8) and *CXCL1*), yielding an M2-like macrophage phenotype [31]. Interestingly, they also observed reduced expression of factors associated with suppression of immune responses, such as indoleamine 2,3-dioxygenase 1 (*IDO1*), which plays an important role in limiting T-cell activation through its metabolism of tryptophan to kynurenine. In coculture experiments with MDMs and autologous T cells, MDMs exposed to PPAR-δ ligands increased CD8^+^/IFNγ^+^ T cell differentiation and limited IDO-1 mRNA and protein expression [31]. In addition, both kynurenine and the checkpoint inhibitor PD-1 ligand were also reduced in PPAR-δ ligand-treated MDMs [31]. Pathway analysis of RNA-Seq data from MDMs revealed upregulation of canonical PPAR-δ target genes involved in lipid metabolism, but exactly how they are engaged in immune regulation remains unclear [31]. Although the reports cited shed some light on the functions of PPAR-δ in immune cells, many questions remain concerning the mechanisms involved. The data reported above suggest that regulation of pro- and anti-inflammatory factor production and/or modulation of sensitivity to inflammatory stimuli may both be important in determining the overall outcome of PPAR-δ activation. The complexity of PPAR-δ’s effects on inflammation is likely to be related to its different modes of function as a transcription factor, which in turn are dependent on specific interactions with target gene sequences (PPREs) as well as the presence or absence of relevant PPAR-δ ligands and coactivators/repressors [67]. 

## 5. The Role of PPAR-δ in Cancer

### 5.1. PPAR-δ Crosstalk between Inflammation and Cancer

The role of chronic inflammation in promoting tumorigenesis is well recognized and considered to be a hallmark of cancer development [68,69]. Colitis-associated colon cancer (CAC) is one of best established examples of chronic inflammation’s role in increasing risk of cancer development, and its effects have been clearly demonstrated in preclinical and clinical studies [69,70]. PPAR-δ’s impact on inflammation-promoted tumorigenesis has been studied by various groups, especially in relation to lipid signaling, as in the case of prostaglandin E_2_ (PGE_2_) [71]. PGE_2_ is an eicosanoid lipid mediator generated through the actions of cyclooxygenases (COX-1 and -2). COX-2/ PGE_2_ signaling is frequently upregulated in tumors and is especially important in the context of inflammation-driven tumorigenesis; it has been particularly well-studied in the case of colonic tumorigenesis [72]. PGE_2_ enhanced PPAR-δ transcriptional activity via PI3 kinase/AKT activation to promote colon cancer cell survival in vitro and intestinal tumorigenesis in APC^Min^ mice [73]. In addition, targeted overexpression of PPAR-δ in intestinal epithelial cells promoted development of azoxymethane (AOM)/dextran sodium sulfate (DSS)-induced CAC in mice via upregulation of IL-6/STAT3 [74]. Activation of PPAR-δ by the synthetic ligand GW501516 in colon cancer cell lines or primary mouse intestinal epithelial cells upregulated COX-2 expression and PGE_2_ production, subsequently increasing macrophage production of proinflammatory cytokines (e.g., CXCL1,CXCL2,CXCL4, and IL-1β) [75]. Both chemically and genetically induced colitis and CAC were markedly suppressed in a PPAR-δ knockout mouse model targeting PPAR-δ’s DNA binding domain (deleting exons 4 and 5), therefore blocking its function as a transcription factor [75]. Considered together, these findings suggest a positive feedback loop between PPAR-δ and COX-2 that orchestrates a proinflammatory microenvironment to enhance tumorigenesis. However, in studies using a different PPAR-δ knockout mouse model targeting the C-terminal portion of the protein (*Ppard* exon 8), female KO mice treated with DSS showed significant increases in some clinical colitis scores (weight loss and colon length) as well as levels of IFN-γ, TNF-α, IL-6, and worsened histopathological scores compared to WT mice [76,77]. These discordant data have been suggested to be secondary to the *Ppard* exon 8 genetic deletion strategy producing a hypomorphic PPAR-δ protein with some retained/altered function as opposed to a complete loss of function [75]. Interestingly, prostatic-epithelial-targeted genetic deletion of PPAR-δ’s DNA binding domain [7] has been recently reported to increase cellularity; in this setting, PPAR-δ was proposed to suppress prostate cancer via DNA-binding-dependent but ligand-binding-independent mechanisms [78]. 

PPAR-δ’s promotion of inflammation and tumorigenesis is not limited to the colon. Tumorigenesis repurposes various components of the inflammatory machinery to create a microenvironment conducive to tumor growth. For example, PPAR-δ is upregulated in tumor-associated macrophages (TAMs) in ovarian-cancer-associated ascites [79], and additional work has shown that macrophages associated with ovarian cancer tend to exhibit protumorigenic properties (e.g., immunosuppression, growth promotion) [80]. In a mouse model of breast cancer, mammary-epithelium-targeted PPAR-δ overexpression promotes tumorigenesis, which is further augmented by treatment with the PPAR-δ agonist GW501516 [81]. Tumor development in this model is associated with upregulation of proinflammatory genes, including COX-2, and activation of AKT signaling [81], reminiscent of the positive association between PPAR-δ, COX-2, and AKT signaling in colorectal tumorigenesis as discussed above. Likewise, a similar positive feedback loop between PPAR-δ and COX-2 signaling has also been demonstrated to enhance the survival of hepatocellular carcinoma cell lines [82]. 

Overall, while the genetic PPAR-δ deletion models generated via targeting exons encoding the PPAR-δ C-terminal region versus the DNA binding domain have yielded discordant data, the preponderance of evidence supports a model in which PPAR-δ strongly enforces inflammation-driven promotion of tumorigenesis through the enhancement of proinflammatory and protumorigenic mechanisms, especially as illustrated in the case of the positive feedback between PPAR-δ and COX-2 (Figure 1). 

### 5.2. PPAR-δ Promotion of Cancer

While PPAR-δ’s relationship to cancer remains controversial [5,83], as more data continue to be published, we should see better clarification of PPAR-δ’s specific contributions to the tumorigenic process. PPAR-δ is overexpressed in various human cancers, including colorectal cancer (CRC) [84,85,86], where it can be upregulated even in early stages (e.g., in adenomas) [84]. Similarly, PPAR-δ is upregulated in other human malignancies, including pancreatic cancer, where its upregulation is correlated with higher pathological grade and increased risk of metastasis [87]. PPAR-δ is also known to be expressed in human lung cancer [88]. Of interest, while PPAR-δ protein expression as assessed by immunohistochemistry has only been observed in the nuclei of normal cells, it becomes nuclear and cytoplasmic in cancer cells [85,86]. The significance of this shift in PPAR-δ distribution in relation to its function is unknown. Generally, high PPAR-δ expression in human cancers is associated with negative survival outcomes [67,86]. 

The controversy regarding PPAR-δ’s role in tumorigenesis primarily stems from preclinical studies as illustrated by the following examples. First, a study using a PPAR-δ knockout mouse model (c-terminal KO/exon 8) showed that *Ppard* germline deletion increased the formation of colon tumors when the mice were bred with APC^Min^ mice or treated with AOM [89]. Later, a study by another group showed opposite results; in this case, *Ppard* germline deletion (DNA binding domain KO/exons 4–5) reduced the formation of colon tumors when the mice were bred with APC^Min^ mice [90]. Studies by our group showed that *Ppard* deletion genetically targeted to the intestinal epithelium profoundly inhibited AOM-induced colonic tumorigenesis [91]. While informative, the clinical relevance of using PPAR-δ knockout models of colorectal tumorigenesis in which PPAR-δ expression is reduced to levels below constitutive levels in normal cells is limited because PPAR-δ is typically upregulated in human colorectal cancer [11,84,86,92]. Modeling PPAR-δ’s influence on CRC by targeting its overexpression to the intestinal epithelial cells better simulated its upregulation in human colon cancer tissues and allowed clear demonstration that PPAR-δ overexpression strongly promoted AOM-induced colorectal tumorigenesis [93].

Interestingly, when PPAR-δ knockout mice with the c-terminal (exon 8) deletion, which led to increased colonic tumorigenesis in the initial study [89], were backcrossed to MMTV-COX-2 transgenic mice on the FVB/N background, COX-2-induced mammary tumorigenesis was markedly suppressed [94]. Furthermore, subsequent studies in which syngeneic PPAR-δ-expressing B16-F10 melanoma cells or Lewis lung cancer cells were implanted into the same PPAR-δ knockout mouse model also showed inhibition of tumorigenesis [95]. These contradictory findings using the same PPAR-δ knockout construct have been interpreted as suggesting that PPAR-δ has different roles depending on where it is expressed—specifically, that PPAR-δ expressed in noncancerous cells in the tumor microenvironment promotes tumorigenesis, whereas PPAR-δ expressed in cancer cells suppresses tumorigenesis [95]. However, in experiments where B16-F10 mouse melanoma cells in which PPAR-δ was downregulated using shRNA were subcutaneously injected into syngeneic (C57BL/6) WT or PPAR-δ germline knockout mice [74], PPAR-δ downregulation in either cancer or noncancer cells inhibited metastasis, although this effect was stronger when PPARδ expression was suppressed in cancer cells [86]. In addition, downregulation of PPAR-δ expression in cancer cells strongly suppressed metastases in orthotopic injection mouse models of multiple human cancers (e.g., colon, lung, melanoma, breast, and pancreas) via downregulation of important prometastatic genes (e.g., *NRG1*, *CXCL8* (encoding IL-8), and *STC1*) in cancer cells and suppression of critical metastatic events including angiogenesis, epithelial-mesenchymal transition (EMT), and cancer cell invasion and migration [86] (Figure 1). PPAR-δ upregulation in human colon, lung, and breast cancers is also correlated with reduced metastasis-free survival [86]. PPAR-δ has also been reported to promote progression of melanoma via Snail expression [96] and prostate cancer via tumorigenic redirection of transforming growth factor β1 (TGF-β1) signaling [97]. A recently published study using unbiased global transcriptome analysis identified PPAR-δ activation as a driver of intestinal stem cell transformation and tumor promotion in APC^Min^ mice maintained on a high-fat diet, suggesting it may play a mechanistic role in obesity-driven cancers [98]. Overall, a scenario is emerging in which PPAR-δ significantly contributes to both the initiation and progression of tumorigenesis in multiple tissues/tumor types. 

## 6. Conclusions

PPAR-δ is a transcription factor that profoundly influences important cellular functions regulating metabolism and inflammation. PPAR-δ’s ability to act as a metabolic switch, shifting cellular energy utilization from glycolysis to fatty acid β-oxidation and thereby improving systemic glycemic control and lipid metabolism, make it an attractive target for prevention or treatment of metabolic-syndrome-related diseases (e.g., obesity, dyslipidemia, diabetes). Nevertheless, emerging data suggest that these same PPAR-δ-triggered mechanisms that help normal cells endure environmental metabolic challenges can also be exploited by cancer cells to promote their survival and, ultimately, cancer progression (Figure 1). Therefore, future therapeutic agents targeting PPAR-δ activation to treat metabolic diseases must be carefully designed and evaluated for any potential risks and off-target effects, including unintended promotion of preneoplastic or neoplastic lesions. Furthermore, our current understanding of the roles of PPAR-δ’s interactions with its endogenous ligands, lipid transporters, and other nuclear receptors, coactivators, and repressors remains incomplete. While some exciting discoveries have been made, more studies are still needed to better define the roles and mechanistic actions of PPAR-δ in different physiological and pathophysiological conditions.

## Abbreviations

AKT	Cellular homolog of viral *akt* gene, also known as protein kinase B
AOM	azoxymethane
APC	Adenomatous polyposis coli
BCL-6	B cell lymphoma 6
CAC	colitis-associated cancer
CRC	Colorectal cancer
COX-2	cyclooxygenase-2
CXCL1	Chemokine (C-X-C motif) ligand 1
CXCL2	Chemokine (C-X-C motif) ligand 2
CXCL4	Chemokine (C-X-C motif) ligand 4
DSS	dextran sodium sulfate
EAE	experimental autoimmune encepaholmyelitis
EMT	Epithelial mesenchymal transition
FABP	Fatty acid binding protein
FAT	Fatty acid translocase
FATP	Fatty acid transport protein
IDO-1	indoleamine 2,3-dioxygenase 1
IFN-γ	Interferon-γ
IL-1β	Interleukin-1β
IL-6	Interleukin-6
IL-8	Interleukin-8
MCP	monocyte chemoattractive protein
MDM	monocyte-derived macrophage
MSC	mesenchymal stem cell
MMTV	Mouse mammary tumor virus
NRG1	Neuregulin 1
NF-κB	Nuclear factor kappa-light-chain-enhancer of activated B cells
PGE_2_	prostaglandin E_2_
PI3K	phosphatidyl inositol 3 kinase
PPRE	PPAR response element
PUFA	polyunsaturated fatty acid
RXR	retinoid X receptor
STAT1/3	Signal Transducer and Activator of Transcription 1/3
STC1	Stanniocalcin 1
TAM	tumor-associated macrophage
TGF-β1	transforming growth factor-β 1
TNF-α	tumor necrosis factor α
